# The antibody loci of the domestic goat (*Capra hircus*)

**DOI:** 10.1007/s00251-017-1033-3

**Published:** 2017-10-23

**Authors:** John C. Schwartz, Rebecca L. Philp, Derek M. Bickhart, Timothy P. L. Smith, John A. Hammond

**Affiliations:** 1 The Pirbright Institute, Pirbright, Surrey GU24 0NF UK; 2 Boyd Orr Centre for Population and Ecosystem Health, Institute of Biodiversity, Animal Health and Comparative Medicine, College of Medical, Veterinary and Life Sciences, University of Glasgow, Glasgow, G12 8QQ UK; 3 Cell Wall Biology and Utilization Research, USDA-ARS, Madison, WI 53706 USA; 4 Meat Animal Research Center, USDA-ARS, Clay Center, NE 68933 USA

**Keywords:** B cell receptor, Kappa light chain, Lambda light chain, Heavy chain

## Abstract

The domestic goat (*Capra hircus*) is an important ruminant species both as a source of antibody-based reagents for research and biomedical applications and as an economically important animal for agriculture, particularly for developing nations that maintain most of the global goat population. Characterization of the loci encoding the goat immune repertoire would be highly beneficial for both vaccine and immune reagent development. However, in goat and other species whose reference genomes were generated using short-read sequencing technologies, the immune loci are poorly assembled as a result of their repetitive nature. Our recent construction of a long-read goat genome assembly (ARS1) has facilitated characterization of all three antibody loci with high confidence and comparative analysis to cattle. We observed broad similarity of goat and cattle antibody-encoding loci but with notable differences that likely influence formation of the functional antibody repertoire. The goat heavy-chain locus is restricted to only four functional and nearly identical *IGHV* genes, in contrast to the ten observed in cattle. Repertoire analysis indicates that light-chain usage is more balanced in goats, with greater representation of kappa light chains (~ 20–30%) compared to that in cattle (~ 5%). The present study represents the first characterization of the goat antibody loci and will help inform future investigations of their antibody responses to disease and vaccination.

**Electronic supplementary material:**

The online version of this article (10.1007/s00251-017-1033-3) contains supplementary material, which is available to authorized users.

## Introduction

Antibodies are critical constituents of adaptive humoral immunity in vertebrates. In mammalian species, antibodies are encoded by three distinct, but closely related genetic loci: the immunoglobulin (IG) heavy-chain (IGH) locus and the kappa (IGK) and lambda (IGL) light-chain loci. The IGH, IGK, and IGL loci each contain multiple variable (V), diversity (D, only in IGH), and joining (J) genes, which undergo recombination during immune cell maturation to generate a highly diverse combinatorial repertoire (Lefranc 2014). The number of V, D, and J genes is variable between species, and some species also have multiple V gene subgroups as a means to increase repertoire diversity. Humans, for example, have 123 to 129 *IGHV* genes of which 38 to 46 are functional and belong to seven functional *IGHV* subgroups (Matsuda et al. 1998; Pallares et al. 1999; Watson Corey et al. 2013). In contrast, cattle have only one functional subgroup containing ten functional *IGHV* genes (Niku et al. 2012; Sinclair et al. 1997). Functional diversity is maintained in cattle by an increased occurrence of sequence changes during recombination, a substantially increased average length of the heavy-chain complementarity-determining region (CDR) 3, and a small number of antibodies with an ultra-long CDR3 resulting from the usage of an exceptionally long *IGHD* gene (Berens et al. 1997; Ma et al. 2016; Pasman et al. 2017; Wang et al. 2013).

The tandem arrays of V, D, and J genes within the antibody loci have evolved by a process of duplication and subsequent diversification, resulting in closely related repeating sequences up to a few kilobases in length. This repetitive nature makes the characterization of antibody loci notoriously challenging. Until very recently, whole genome sequencing projects have relied on short-read sequencing with read lengths of 75 bp to 1 kb. The ability to assemble genome sequences across repetitive regions is difficult or impossible if the reads are shorter than the length of the repeat. The antibody loci in most current genome assemblies are therefore highly fragmented, incorrectly assembled, contracted due to repeats piling up at the same position during automatic assembly, or expanded due to the incorrect incorporation of allelic variants between haplotypes. The use of mate-pair sequencing to span larger distances, which can help to scaffold the assemblies to some extent, has not overcome this limitation in genome assembly projects. As a result, the best characterizations of antibody loci to date have relied on the targeted sequencing of bacterial artificial chromosomes (BACs) containing antibody sequence. Even though BAC clones are relatively short (~ 100–200 kb) and represent a single haplotype, the use of short reads in sequencing these clones cannot fully surmount the difficulties in assembling the germline sequence.

The IGH, IGK, and IGL loci have been characterized in a few species within the *Cetartiodactyla*, a mammalian clade which includes many agriculturally important and model species. The pig (*Sus scrofa*) is perhaps the best characterized of these. Much of the pig IGH locus has been characterized from overlapping BAC sequences (Eguchi-Ogawa et al. 2012; Eguchi-Ogawa et al. 2010), as have the IGK and IGL loci (Guo et al. 2016; Schwartz et al. 2012a; Schwartz et al. 2012b; Schwartz and Murtaugh 2014). Results of these studies were consistent with analyses of porcine antibody repertoire and B cell development, reviewed extensively by Butler et al. (2017). Ruminants, such as cattle (*Bos taurus*), sheep (*Ovis aries*), and goats (*Capra hircus*), diverged from pigs approximately 60 Mya (Meredith et al. 2011) and have evolved notable differences with respect to their antibody repertoires. For example, cattle B cells almost exclusively express lambda light chains, whereas pig B cells express lambda and kappa at approximately equal frequency (Arun et al. 1996; Butler et al. 2005; Hood et al. 1967; Sinkora et al. 2001). Cattle exclusively express a completely different *IGHV* subgroup compared to pigs and rely on only a single *IGLV* subgroup instead of two or three as in pigs. It should be noted that the antibody loci of cattle have largely been characterized using the available reference genome assemblies, which are fragmented into many contigs (Ekman et al. 2009; Niku et al. 2012; Pasman et al. 2010), and only very recently was the cattle IGH characterized using overlapping BAC clones (Ma et al. 2016). In sheep, the heavy-chain locus in the genome assembly (Oar_v3.1) is heavily disrupted and mostly missing so that only the light-chain loci are reasonably characterized (Qin et al. 2015).

Goats diverged from cattle approximately 30 Mya (Hiendleder et al. 1998) and from sheep approximately 15 Mya (Nomura et al. 2013). However, despite the economic importance of goats in both agriculture and for the production of antibody-based reagents, surprisingly little is known about the goat antibody repertoire. The San Clemente goat is a highly homozygous domesticated breed that was recently used to create the most contiguous genome assembly for a non-model organism reported to date (Bickhart et al. 2017). The assembly process used a combination of technologies including long-read Pacific Biosciences (PacBio) single molecule real-time sequencing, physical mapping methods, and error correction using shorter Illumina reads. The resulting assembly had greatly improved representations of repetitive immune complexes, including the antibody loci, which were mostly intact relative to the previous goat reference assembly, CHIR_2.0 (Bickhart et al. 2017). As such, we sought to more completely characterize the three antibody loci in the domestic goat and compare them to what is known in other related species, such as cattle, sheep, and pigs.

## Materials and methods

### Ethics statement

Peripheral blood samples from goats and cattle were collected in accordance with the UK Animal (Scientific Procedures) Act, 1986, and approved by The Pirbright Institute Animal Welfare Ethical Review Board.

### Genomic sequence, annotation, and nomenclature

The long-read goat genome sequence assembly (Bickhart et al. 2017) was deposited in GenBank (BioProject accession: PRJNA290100). The IGH locus is contained on the scaffold for chromosome 21 (GenBank: NC_030828.1) and nine additional unplaced contigs (GenBank: LWLT01000331, LWLT01000361, LWLT01000364, LWLT01000390, LWLT01008200, LWLT01000862, LWLT01008318, LWLT01001482, and LWLT01010025). The IGL locus is on chromosome 17 (GenBank: NC_030824.1), and the IGK locus is on chromosome 11 (GenBank: NC_030818.1). These regions were manually annotated for antibody gene features using Artemis (Rutherford et al. 2000). Sequence similarity to the known cattle V, D, and J genes and light-chain constant regions (Ekman et al. 2009; Ma et al. 2016; Pasman et al. 2010) was used to predict the related genes in the goat using the basic local alignment search tool (BLAST) (Altschul et al. 1990). The identification of more divergent V genes was further aided by searching for immunoglobulin domains using CD-search (Marchler-Bauer and Bryant 2004) and comparing the resulting sequences to the IMGT database (Giudicelli et al. 2005). Pseudogenes were defined based on the presence of a truncation, nonsense mutation, frameshift, or defective initiation codon, any of which would prevent the production of a functional protein. All alignments were generated using CLUSTALW with default parameters (Thompson et al. 1994). Genes were named and amino acid positions were numbered according to the IMGT nomenclature (Lefranc 2007, 2011; Lefranc et al. 2003). Subgroup or clan designations were based on sequence similarity to known V genes in other species using the IMGT/V-QUEST (Brochet et al. 2008).

All goat *IGHV* genes were found to be most closely related to the human *IGHV2*, *IGHV3*, and *IGHV4* subgroups using the IMGT/V-QUEST (number of aligned reference sequences 15; advanced parameters with allele *01 only), which is consistent with cattle and sheep. All four functional goat *IGHV* genes belong to the ruminant *IGHV1* subgroup and are approximately 72 to 73% identical to the human *IGHV4* subgroup. Thus, the existing IMGT nomenclature system for ruminants was used to classify the goat *IGHV* genes to the ruminant *IGHV1*, *IGHV2*, and *IGHV3* subgroups. The *IGHG1*, *IGHG2*, and *IGHG3* constant genes were named based in their similarity and likely orthology with those in cattle. Sheep sequences for *IGHG* were acquired from GenBank (*IGHG1*, accession: X69797; *IGHG2*, accession: X70983) or from the sheep reference assembly Oar_v3.1 (*IGHG3*, chr. 18: 68,549,650–68,551,242) using the Ensembl genome browser (Cunningham et al. 2015).

### Animals and RNA isolation

Four apparently healthy Friesian cattle, aged 30 months, were selected from a herd at The Pirbright Institute. Additionally, four female Saanen goats, aged 6 months, were selected from a healthy commercial herd in the UK. Peripheral blood was collected by venipuncture into heparinized tubes, and mononuclear cells (PBMCs) were isolated by density gradient cell separation using Histopaque-1077 (Sigma-Aldrich), and contaminating erythrocytes were lysed in ammonium chloride lysis buffer (160 mM ammonium chloride, 170 mM Tris, pH 7.65). PBMCs were mixed with TRIzol (Thermo Fisher Scientific) and chloroform, and total RNA was extracted from the aqueous phase and purified using isopropanol and ethanol precipitations according to the manufacturer’s guidelines. Complementary DNA (cDNA) was synthesized using oligo(dT)_12–18_ primers and the SuperScript II reverse transcriptase kit (Thermo Fisher Scientific) according to the manufacturer’s protocol and quantified using a Qubit fluorometer (Thermo Fisher Scientific).

### Relative quantification of light-chain transcripts

Oligonucleotide primers were designed to amplify *IGKC* and *IGLC* as well as the housekeeping genes *PPIA*, *SDHA*, and *ACTB* from both cattle and goats with product sizes of approximately 120 bp (Supplementary Table 1). Specific amplification for each primer pair was confirmed using Sanger sequencing on PCR products derived from multiple animals. Quantitative real-time PCR (qPCR) reactions were performed in triplicate with the isolated cDNA using the Luminaris Color HiGreen Low ROX qPCR master mix (Thermo Fisher Scientific) and the QuantStudio 5 (Applied Biosystems) platform. Reactions contained 10 ng cDNA, 1× master mix, and 300 nM of each primer in a final volume of 20 μl. Thermal cycling conditions were as follows: 50 °C for 2 min, 95 °C for 5 min, followed by 40 cycles at 95 °C for 15 s, 58 °C for 1 min, and 72 °C for 45 s. Dissociation curves were also generated for each reaction product using incremental heating steps from 58 to 95 °C to further assess specificity. The qbase^+^ software (Biogazelle NV, Belgium) was used for qPCR sample comparisons with *PPIA*, *SDHA*, and *ACTB* as reference genes and positive controls. The cycle threshold (Ct) values were used to calculate an expression ratio between *IGKC* and *IGLC* for each individual animal. These calculations were then used to perform a one-tailed Wilcoxon rank-sum test on the hypothesis that the distribution of *IGKC* expression is increased in goats compared to cattle. This test was chosen instead of the *t* test as it does not assume normality.

## Results

### The goat Ig heavy-chain locus

**Fig. 1:**
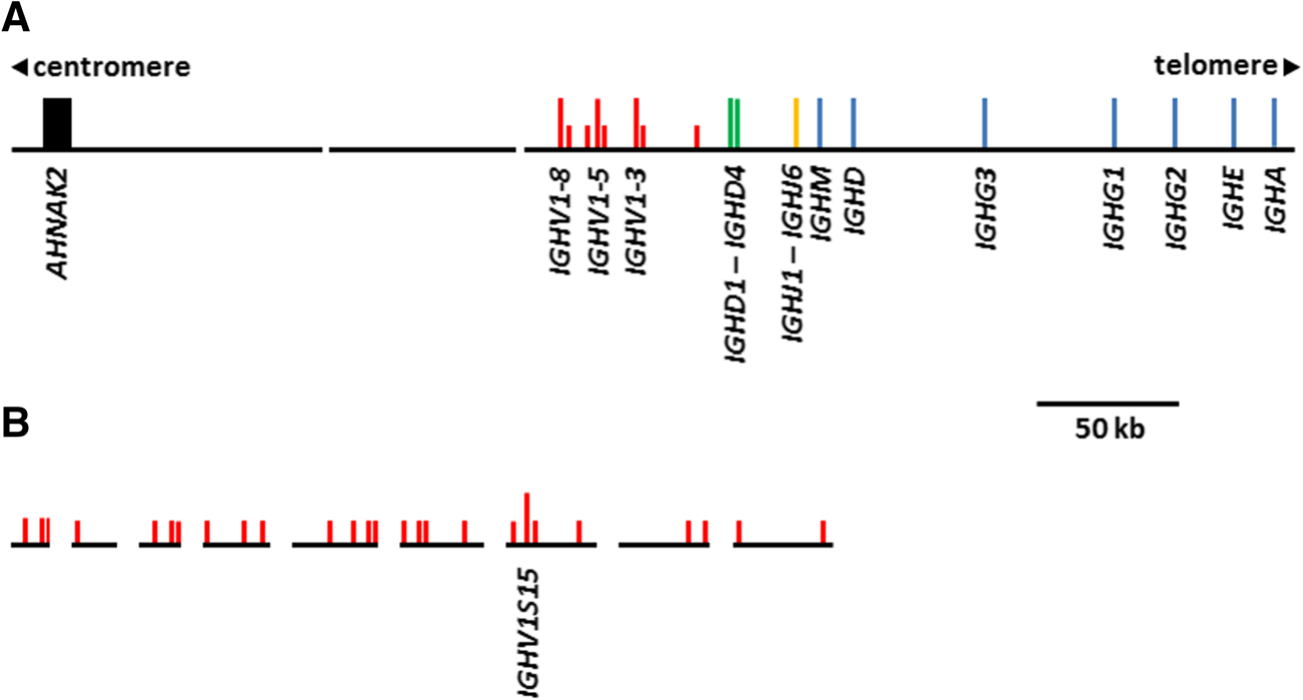
Organization of the goat antibody heavy-chain locus on the chromosome 21 scaffold (**a**) and unplaced contigs (**b**). Vertical bars indicate the position of individual genes or exons. Long vertical bars indicate putatively functional genes, whereas short bars indicate putatively non-functional genes. Only functional genes are labeled. Sequence gaps are indicated by breaks in the horizontal backbone. *Red*, V gene; *green*, D gene; *orange*, J gene; *blue*, constant region; *black*, other. The contigs shown in (**b**) are in the following order from left to right (GenBank accessions): LWLT01010025, LWLT01008318, LWLT01008200, LWLT01001482, LWLT01000862, LWLT01000390, LWLT01000364, LWLT01000361, LWLT01000331. Of these, the second, third, sixth, seventh, and ninth have been inverted to make their orientation consistent with the other contigs

The goat heavy-chain locus is found on chromosome 21q24 (Schibler et al. 2009). Our previous analyses revealed that this region remains partially fragmented in the ARS1 assembly, possibly because the sequence data was generated from whole blood cell DNA (Bickhart et al. 2017). However, it is substantially improved compared to the previous reference assembly, CHIR_2.0. Ten contigs spanning the IGH locus were identified in the ARS1 assembly. Of these contigs, the bulk of the locus is contained on a single 275 kb contig on the chromosome 21 scaffold (Fig. 1a), whereas the remaining nine contigs comprise approximately 219 kb of unplaced sequence (Fig. 1b). Our analyses identified 34 *IGHV* genes across all ten contigs (Fig. 1a, b and Supplementary Table 2). Of the 34 *IGHV*, only four are putatively functional, and these are all very similar to each other (Supplementary Fig. 1), indicating a lack of functional germline diversity. The remaining 30 non-functional *IGHV* genes are typically heavily disrupted by frameshifts, premature stop codons, truncations, and disrupted promoter sequences and splice site junctions (Supplementary Table 2). Several of the unmapped *IGHV* genes appear to be duplicated (*IGHV1S11*/*IGHV1S19*, *IGHV3S12*/*IGHV3S20*, and *IGHV2S17*/*IGHV2S30*), although it is uncertain if this is due to the presence of nearly identical duplications in the genome or the incorporation of sequence from both chromosomes into the assembly. As in cattle, the goat *IGHV* genes belong to three ruminant-specific *IGHV* subgroups, and like cattle, the *IGHV2* and *IGHV3* subgroup genes are all non-functional (Niku et al. 2012). Consequently, the goat IGH repertoire is severely restricted at the germline level, suggesting that heavy-chain diversity in this species must be largely driven by post-recombinatorial mechanisms, such as somatic gene conversion and/or somatic hypermutation.

The 275 kb contig placed with chromosome 21 contains the entirety of the constant region, four *IGHD* genes (Supplementary Fig. 2), six *IGHJ* genes (Supplementary Fig. 3), and the first eight *IGHV* genes. In contrast to cattle, there is no evidence that goats have duplicated the *IGHD*, *IGHJ*, and *IGHC* genes (Ma et al. 2016). As a result, goats lack the ultra-long *IGHD* gene as seen in cattle (Ma et al. 2016; Wang et al. 2013). We previously noted that the flanking gene *AHNAK2* is found upstream from the *IGHV* region in the new goat ARS1 assembly, whereas in humans, this gene is downstream from the IGH constant region. This structure in the goat was supported by three radiation hybridization probes (Bickhart et al. 2017), indicating that the goat IGH locus is inverted relative to humans.

**Fig. 2:**
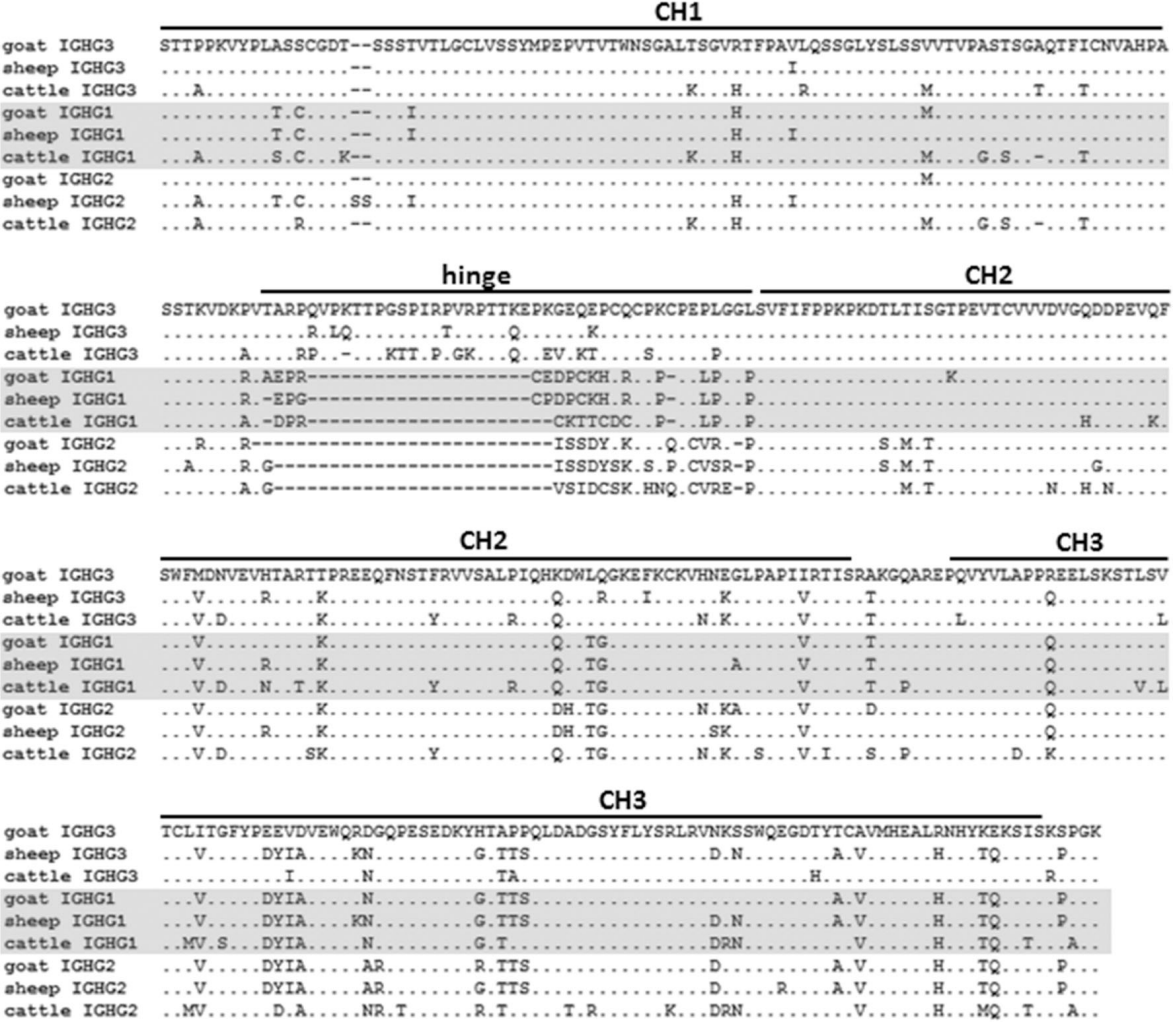
Alignment of putative amino acid sequences for the *IGHG* subclasses in ruminants. *IGHG* subclasses are separated by *gray shading*. Cattle sequences are derived from a genomic BAC construct (GenBank: KT723008) as recently published (Ma et al. 2016). Sheep *IGHG1* and *IGHG2* are based on the mRNA GenBank accession sequences X69797 and X70983, respectively. Sheep *IGHG3* is based on genomic sequence found in the Oar_v3.1 genome assembly contig AMGL01043778, acquired from the Ensembl genome browser and manually annotated

**Fig. 3:**
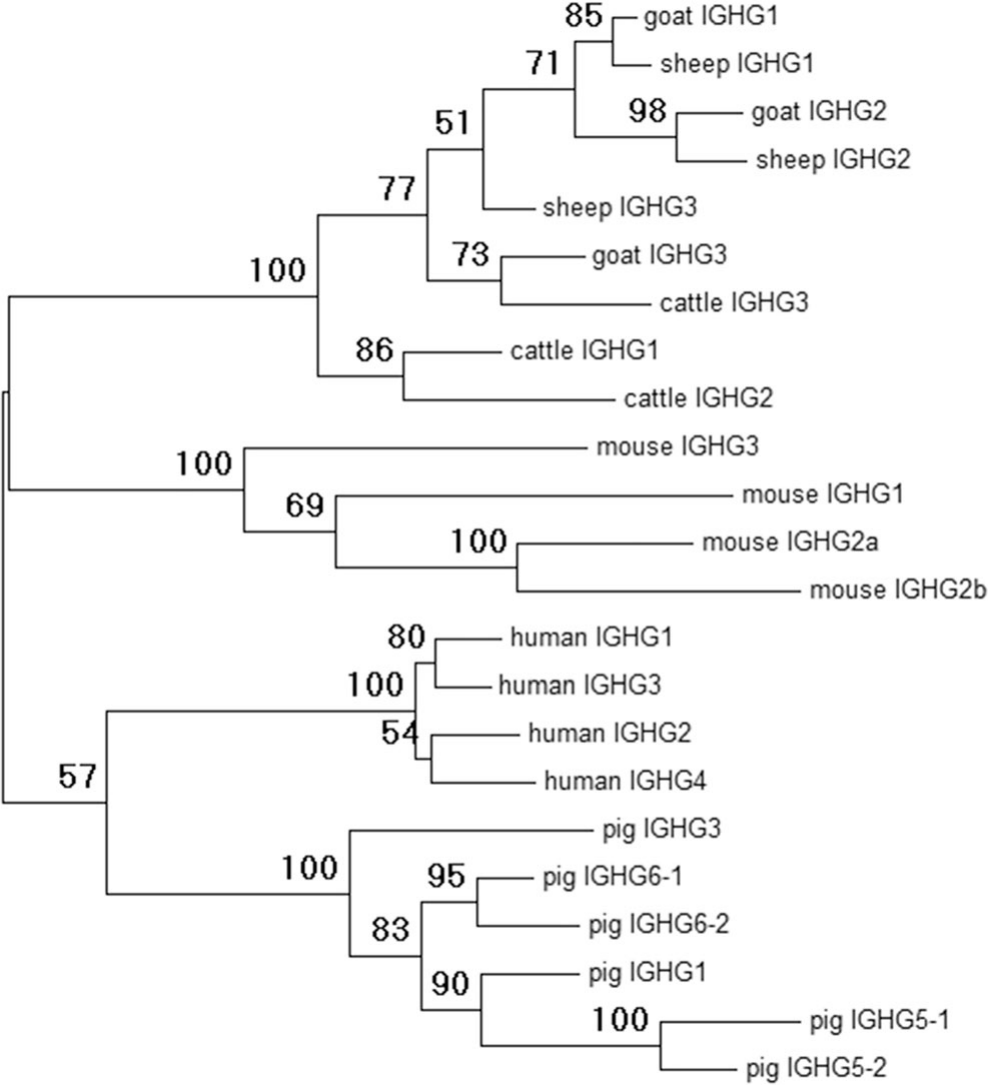
Phylogenetic analysis of putative IGHG amino acid sequences from goats, sheep, cattle, mice, humans, and pigs. Amino acid sequences for goats, sheep, and cattle were acquired as described in Fig. 2. Pig sequences were acquired from either of two genomic BAC constructs (GenBank accession: AB699686 for *IGHG1*, *IGHG3*, *IGHG5-1*, and *IGHG5-2*; AB699687 for *IGHG6-1* and *IGHG6-2*) as previously described (Eguchi-Ogawa et al. 2012). Mouse sequences were acquired from a genomic contig (GenBank: D78344) as previously published (Akahori and Kurosawa 1997). Sequence for human *IGHG1* was acquired from the genome assembly for chromosome 14 (GenBank: NC_000014.9). The remaining human sequences were acquired from cDNA (*IGHG2*, AJ250170; *IGHG3*, AJ390284; and *IGHG4*, AJ294733). The tree was generated using maximum likelihood based on the Jones-Taylor-Thornton model (Jones et al. 1992) and 100 bootstrap iterations within MEGA6 (Tamura et al. 2013). Nodal bootstrap values are indicated

Goats have three constant region *IGHG* subclasses (Fig. 1a), as in cattle and sheep. Although the three immunoglobulin-like heavy-chain constant (CH) domains are all very similar to each other, the three subclasses differ greatly in the hinge region (Fig. 2), which reflects their differential abilities to interact with various Fc receptors. Phylogenetic analysis confirmed that these three ruminant subclasses also clade together when compared to pigs, mice, and humans (Fig. 3). However, among ruminants, they do not consistently clade with each other or with their respective species. This is most likely due to sequence similarity across the CH domains, which appear to have homogenized so that they appear more similar within each species (Fig. 2). Together, these data confirm that the goat *IGHG* subclasses share common ancestry with cattle and sheep and are likely functionally equivalent between these species.

### The goat Ig lambda light-chain locus

**Fig. 4:**
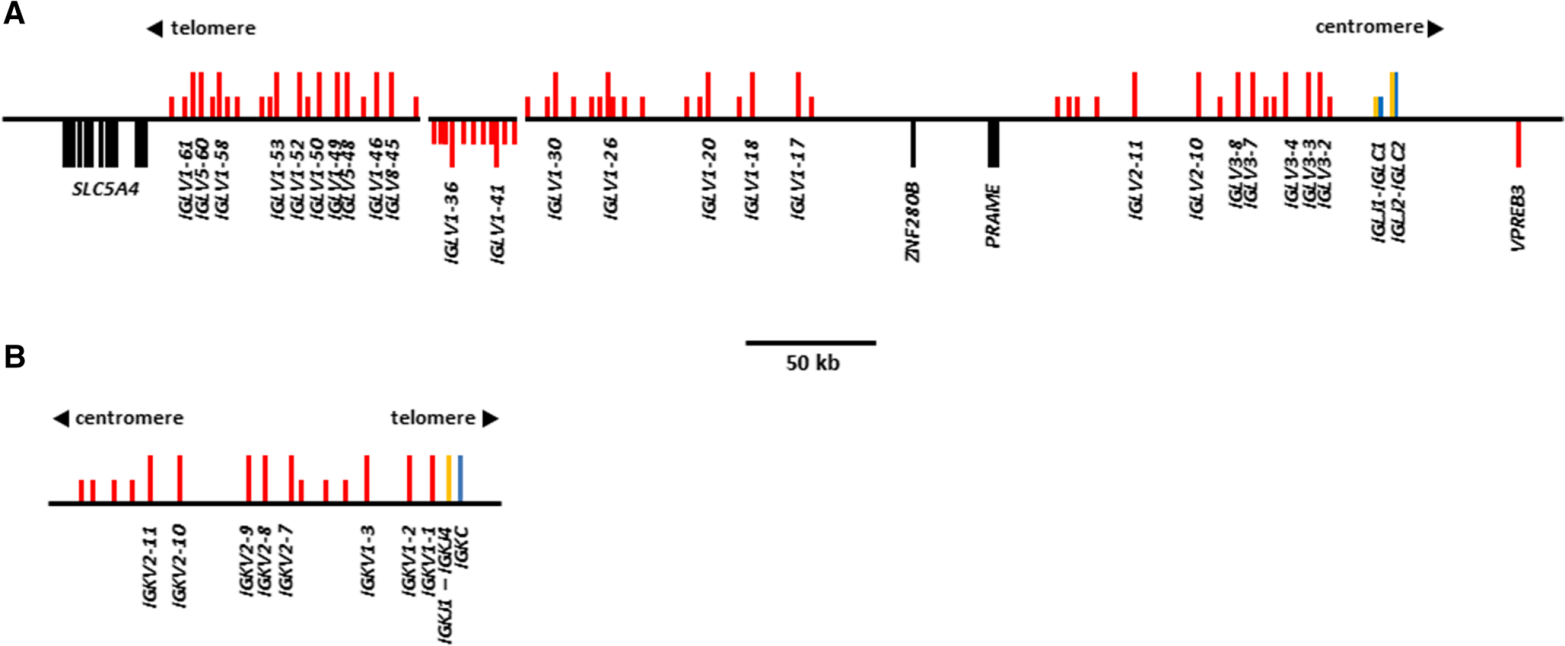
Organization of the antibody light-chain lambda (**a**) and kappa (**b**) loci in the domestic goat. Genes are labeled as in Fig. 1. Orientation of vertical bars relative to the horizontal backbone indicates transcriptional orientation. The regions displayed represent the ARS1 genomic scaffolds for chromosome 17: 125,000–725,000 (IGL; GenBank: NC_030824.1) and chromosome 11: 46,750,000–46,925,000 (IGK; GenBank: NC_030818.1)

In ARS1, the IGL locus spans approximately 460 kb and contains two sequence gaps on a single scaffold representing chromosome 17. A contig located in the middle of the *IGLV* region is most likely erroneously inverted relative to the rest of the locus (Fig. 4a) (Bickhart et al. 2017). Our previous analysis of the goat *IGLV* region revealed that a substantial amount of sequence was absent from the CHIR_2.0 reference assembly—a result of the highly repetitive nature of these genes (Bickhart et al. 2017). In ARS1, the *IGLV* genes are separated into two distinct clusters by an 89 kb region containing the genes *ZNF280B* and *PRAME* and flanked on the 5′ end by *SLC5A4*. This organization is consistent with other described species, including cattle and pigs (Pasman et al. 2010; Schwartz et al. 2012b).

The constant region is comprised of *IGLJ*-*IGLC* cassettes, of which there are two in the goat. However, goat *IGLC1* is truncated by a nonsense mutation at IMGT position 41 (Supplementary Fig. 4). This likely results in a non-functional constant region, as it would prevent both the formation of the disulfide bond between the light and heavy chains as well as the internal disulfide bond between C23 and C104. This observation is consistent with sheep, which possess the same nonsense mutation (Qin et al. 2015). Likewise, in both goats and sheep, the associated *IGLJ* gene contains a non-canonical LGGG motif, despite having an intact recombination signal (RS) (Supplementary Fig. 5). Thus, both goats and sheep are functionally restricted to the single remaining *IGLJ-IGLC* cassette. Consequently, IGL-CDR3 diversity in the goat is predicted to be nearly entirely derived from the rearrangeable *IGLV* genes.

A total of 63 *IGLV* genes were identified upstream from the constant region (Fig. 4a). This is equivalent to the number previously reported in the cattle Btau_3.1 assembly (Ekman et al. 2009), yet more than the 43 *IGLV* in sheep (Qin et al. 2015), and more than the 23 *IGLV* in pigs (Schwartz et al. 2012b; Schwartz and Murtaugh 2014). The *IGLV* subgroups are relatively consistent between these species, with those of the *IGLC*-proximal cluster being most similar to the *IGLV2* and *IGLV3* subgroups of humans, and those of the *IGLC*-distal cluster most similar to the *IGLV1*, *IGLV5*, and *IGLV8* subgroups. Of the 63 *IGLV* genes in the goat, 25 are putatively functional and spread across these five subgroups, although 15 of these belong to *IGLV1* and five belong to *IGLV3* (Supplementary Table 3 and Supplementary Fig. 6).

### The goat Ig kappa light-chain locus

The goat IGK locus on chromosome 11 is contained on a single contig (Bickhart et al. 2017). The 5′ end of the locus is flanked by a gene desert with no additional immunoglobulin domains identified within 200 kb upstream of the first *IGKV* gene, indicating that the entire locus is present. The IGK locus is considerably reduced in size compared to the IGL and contains only 15 *IGKV* genes (Fig. 4b), which is also fewer than the 24 *IGKV* reported in cattle (Ekman et al. 2009). However, both goats and cattle possess eight putatively functional *IGKV* (Supplementary Table 4 and Supplementary Fig. 7). Of these functional *IGKV* in the goat, five belong to the *IGKV2* subgroup and the other three to the *IGKV1*. Two of the three *IGKV1* genes possess a non-canonical heptamer in their RS, likely rendering V-J recombination highly inefficient. In contrast, all five *IGKV2* genes possess a canonical RS, suggesting that these are likely represented in the majority of expressed kappa chains.

**Fig. 5:**
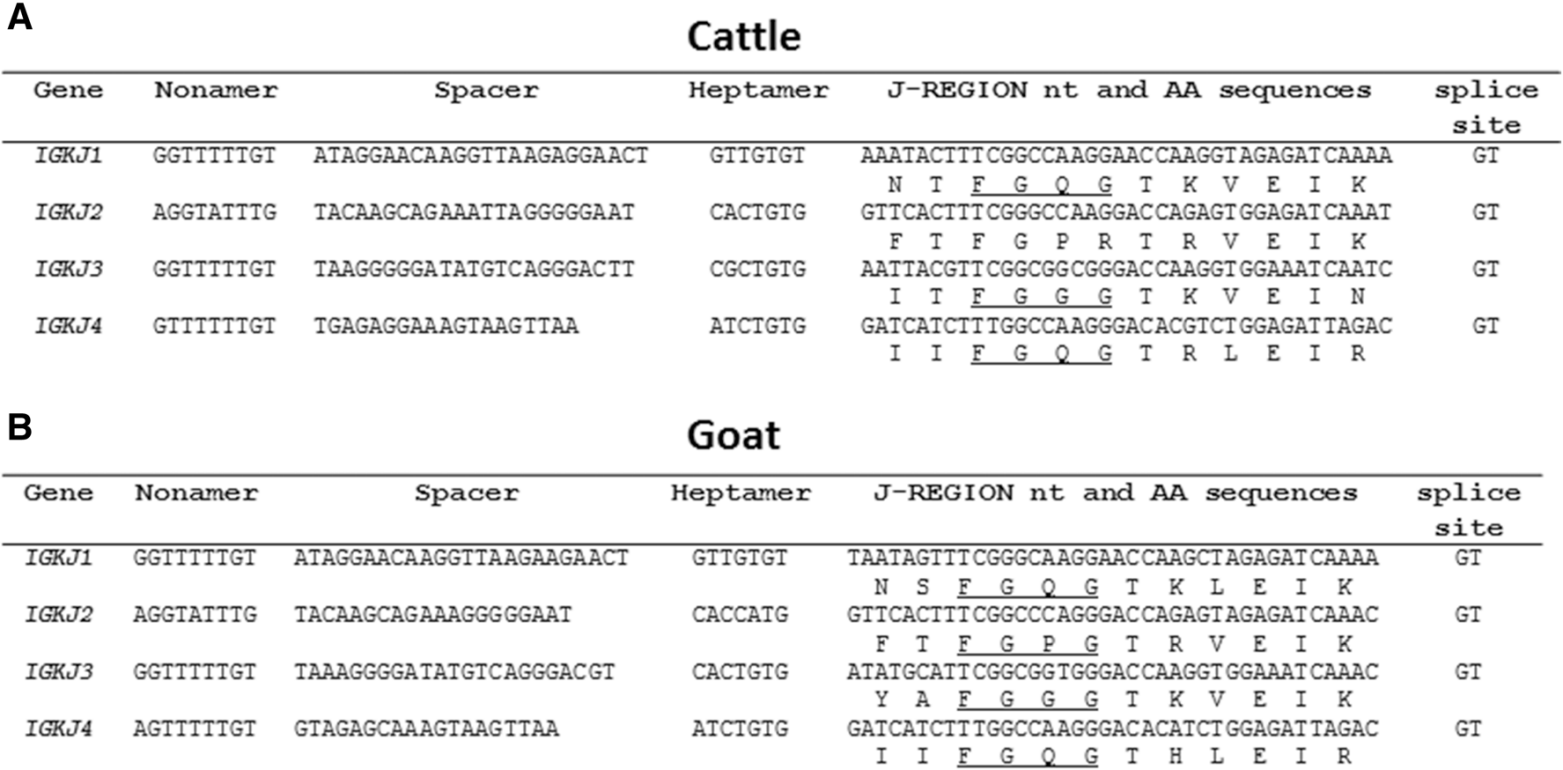
Genomic features of the cattle (**a**) and goat (**b**) *IGKJ* genes. The canonical W/F-G-X-G framework motif is underlined where intact

There are four *IGKJ* genes downstream from the *IGKV* region, one more than previously reported in cattle (Ekman et al. 2009). However, the current cattle reference genome contains a sequence homologous to the fourth goat *IGKJ* in the position implied by conserved synteny, indicating that the *IGKJ* repertoire is highly similar between these two species (Fig. 5a, b). Of the four goat *IGKJ* genes, *IGKJ3* is the only one associated with both a canonical heptamer (i.e. CACTGTG) and a canonical 23-bp spacer within the RS. This strongly suggests that only *IGKJ3* can be efficiently rearranged in the goat (Fig. 5b). We additionally observed from the three published cattle *IGKJ* sequences (as well as the unpublished *IGKJ4*) that only cattle *IGKJ2* possesses a canonical heptamer (Fig. 5a). However, cattle *IGKJ2* is rendered non-functional due to a mutated W/F-G-X-G motif in its framework region (Ekman et al. 2009). As a result, cattle most likely rely on *IGKJ3* which is likely inefficiently rearranged due to its mutated heptamer.

### IGK is more abundantly expressed in goats compared to cattle

**Fig. 6:**
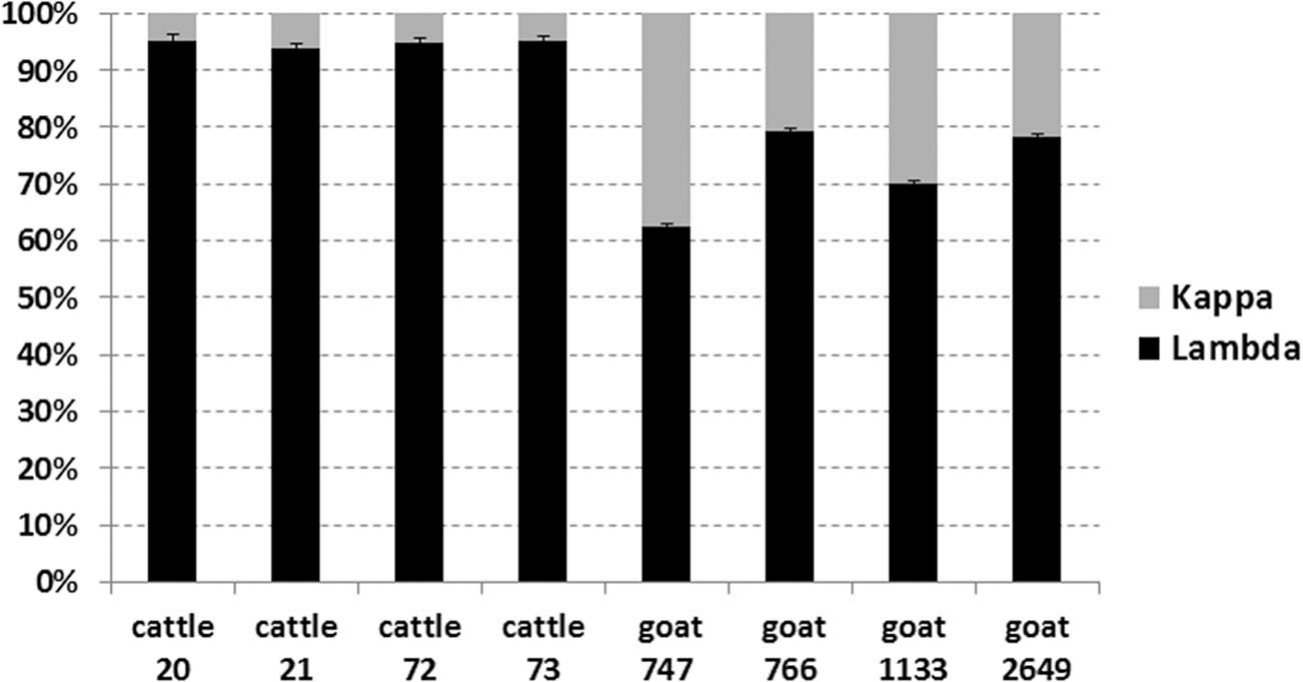
Relative quantification of *IGKC* (*gray*) and *IGLC* (*black*) transcripts in cattle and goat PBMCs from four animals each. Results represent the average of three replicates, and standard error between replicates is shown. The greater expression of *IGKC* in goats is statistically significant (*P* = 0.014286) using a one-tailed Wilcoxon rank-sum test

Because we observed that all of the cattle *IGKJ* sequences possess either a non-canonical RS or a non-functional framework motif and that goats possess a fully functional *IGKJ3* with an intact RS, we hypothesized that the proportional contribution of kappa light chains in the expressed antibody repertoire is higher in goats relative to cattle. We therefore tested the relative contribution of *IGLC* and *IGKC* in both goat and cattle PBMCs using qPCR. Our results confirmed that IGK usage in the cattle antibody repertoire is approximately 5% (Fig. 6), which is consistent with previous reports (Arun et al. 1996; Butler et al. 2005; Hood et al. 1967; Sinkora et al. 2001), and approximately 20–35% in goats (Fig. 6). This difference between cattle and goat light-chain usage was statistically significant (*P* = 0.014286) using a Wilcoxon rank-sum test. The single mutation in the heptamer of *IGKJ3* (i.e. CACTGTG > CGCTGTG) likely drives the restriction of IGK in cattle.

## Discussion

### The goat antibody loci and light-chain usage relative to other related species

We have presently characterized the three antibody loci in the domestic goat and, where possible, compared their germline repertoire to related species. Although the *IGHV* region remains disrupted in the new long-read ARS1 assembly, our identification of numerous *IGHV* genes confirms that the goat utilizes the same subgroup of closely related genes as cattle. However, only four nearly identical putatively functional *IGHV* were identified in the goat compared to ten identified previously in cattle (Niku et al. 2012). It is possible given the degree of fragmentation in this region that there are more *IGHV* genes in goats not identified here; although given the completeness of the other antibody loci and the use of both PacBio and Illumina reads to generate and error correct the assembly, it seems unlikely that many would be missing. The degree of fragmentation observed in the IGH locus is quite possibly due to the use of DNA extracted from blood as the sequencing material, since much of this would be from circulating B cells that have rearranged their antibody loci. Furthermore, unlike cattle, goats have not duplicated the *IGHD* or *IGHJ* regions or the *IGHC* region (specifically *IGHD* and *IGHM*) (Ma et al. 2016; Wang et al. 2013). Because of this, goats lack the *IGHD* gene that enables cattle to generate ultra-long heavy-chain CDR3s. The paucity of germline heavy-chain diversity in the goat suggests that this and other species are heavily reliant on junctional diversity, somatic hypermutation, and/or somatic gene conversion for the diversification of its primary heavy-chain repertoire, although detailed studies of repertoire formation are needed to understand these processes.

We identified the same number of *IGLV* genes in the goat (*n* = 63) as previously identified in cattle (Ekman et al. 2009), which is also more than found in sheep (*n* = 43) (Qin et al. 2015). However, both the sheep and cattle IGL characterizations were based on the current reference genome assemblies which are heavily fragmented across the *IGLV* region (Ekman et al. 2009; Qin et al. 2015). It is therefore quite likely that there remain unidentified genes in these species. Indeed, our preliminary analysis of a recent long-read assembly in cattle indicates that there are many additional unreported *IGLV* genes in that species (unpublished).

The goat IGK locus is substantially reduced in size and complexity relative to the IGL. Although a similar arrangement exists in cattle, we found that goats utilize IGK more than cattle (~ 20–30 vs ~ 5%, respectively) but less than pigs which use IGK in approximately half of all antibodies (Arun et al. 1996; Butler 1983; Butler et al. 2005; Hood et al. 1967; Sinkora et al. 2001). Nevertheless, the ruminant IGK locus appears to be no less complex than in pigs which possess ≥ 9 functional *IGKV* and five functional *IGKJ*, although only one of these *IGKJ* has a canonical RS (Schwartz et al. 2012a). Goats, cattle, and pigs all possess intact kappa enhancers, a kappa-deleting element, and an *IGKJ*-*IGKC* intronic RS heptamer (Das et al. 2009). This, combined with the lack of canonical RS heptamers in the functional cattle *IGKJ* genes suggests that in cattle, recombination may favor either non-functional IGK rearrangements or IGK ablation via recombination with the intronic heptamer and/or the kappa-deleting element downstream from the *IGKC*. In either case, this results in a substantially reduced expressed IGK repertoire in cattle. The abundance of goat light-chain genes, the relative lack of functional IGH genes, and the greater usage of IGK in goats indicate that the potential light-chain diversity in the available repertoire is greater in goats than in cattle, while germline IGH diversity is reduced. The comparison of the expressed (i.e. mRNA) antibody repertoire with the genomic sequence is beyond the scope of the present study. Future work is therefore necessary to confirm gene usage and investigate the mechanisms of post-recombinatorial diversification of goat B cells.

### *IGHG* subclasses are predicted to be functionally equivalent between cattle and goats

The *IGHG* constant region subclasses are functionally responsible for a diverse range of responses, which are often subclass-specific. Except between closely related species, such as ruminants, the *IGHG* subclasses have independently expanded and diversified following speciation (Butler and Wertz 2006; Butler et al. 2009; Eguchi-Ogawa et al. 2012). In contrast, early investigations indicated that cattle, sheep, and goat IgG subclasses were very similar to each other based on the cross-reactivity of subclass-specific antisera (Butler 1983). We identified apparent gene conversion-mediated homogenization between the *IGHG* subclasses within the Ig domains, perhaps indicative of selective pressure to maintain similar Ig constant domains between the *IGHG* subclasses. However, the hinge region remained unique between the subclasses and very similar between cattle, goats, and sheep. Because the CH2-proximal portion of the hinge is known to be directly involved in FcγR binding (Chappel et al. 1991; Duncan et al. 1988; Radaev and Sun 2002), the relative conservation of the hinge region of these species’ *IGHG* subclasses strongly suggests that the subclasses are both functionally equivalent between these species and descended from common ancestral genes. Cattle *IGHG2* is known to have a unique Fc receptor (FcR) encoded by *FCG2R* (Zhang et al. 1995) and which appears to play a major role in pathogen opsonization and phagocytosis (Howard 1984). Peculiarly, this receptor is poorly related to the other FcγRs but very closely related to the leukocyte immunoglobulin-like receptors (*LILR*) encoded within the leukocyte receptor complex (LRC) (Morton et al. 1999; Zhang et al. 1995). The conservation of the hinge region in goat *IGHG2* is further consistent with our observations that *FCG2R* is conserved in the goat LRC yet absent from the pig LRC (J.C. Schwartz and J.A. Hammond, unpublished). The current results will enable more detailed studies into goat humoral immunity and B cell development and fill an important evolutionary link in our understanding of the B cell repertoire.

## Electronic supplementary material

ESM 1 (PDF 1194 kb)
