# Supplementary material for: The antibody loci of the domestic goat (Capra hircus)
Source: Immunogenetics. 2017 Oct 23;70(5):317–26. doi: 10.1007/s00251-017-1033-3 (PMC5899754; doi:10.1007/s00251-017-1033-3)
Supplement: Supplementary file 1 — (PDF 1194 kb) [file 251_2017_1033_MOESM1_ESM.pdf]

Supplementary Material

The antibody loci of the domestic goat (*Capra hircus*)

John C. Schwartz[1], Rebecca L. Philp[1, 2], Derek M. Bickhart[3], Timothy P. L. Smith[4], and John A. Hammond[1†]

[1] Livestock Viral Diseases Programme, The Pirbright Institute, Pirbright, Surrey GU24 0NF, UK

[2] Boyd Orr Centre for Population and Ecosystem Health, Institute of Biodiversity, Animal Health and Comparative Medicine, College of Medical, Veterinary and Life Sciences, University of Glasgow, Glasgow G12 8QQ, UK

[3] Cell Wall Biology and Utilization Research, USDA-ARS, Madison, WI 53706, USA

[4] Meat Animal Research Center, USDA-ARS, Clay Center, NE 68933, USA

[†] Corresponding author

Phone: (+44)1483231397

E-mail: john.hammond@pirbright.ac.uk

Table of Contents

Supplementary Table 1 – qPCR primers

Heavy Chain

IGH locus

Supplementary Table 2 – *IGHV* genomic features

Supplementary Figure 1 – *IGHV* protein display

Supplementary Figure 2 – *IGHD* genomic features

Supplementary Figure 3 – *IGHJ* genomic features

Light Chain

Supplementary Figure 4 – *IGLC* and *IGKC* protein display

IGL locus

Supplementary Figure 5 – *IGLJ* genomic features

Supplementary Table 3 – *IGLV* genomic features

Supplementary Figure 6 – *IGLV* protein display

IGK locus

Supplementary Table 4 – *IGKV* genomic features

Supplementary Figure 7 – *IGKV* protein display

Supplementary Table 1 – qPCR primers

**gene**	**orientation**	**sequence (5'-)**
ACTB	F	ACCGTGAGAAGATGACCCAG
ACTB	R	AGGAAGGAAGGCTGGAAGAG
SDHA	F	GCTCTCCTACGTTGACATCA
SDHA	R	AAGCCTCAGTCTCCTCAGTA
PPIA	F	GCATCTTGTCCATGGCAAAT
PPIA	R	TTCATGCCCTCTTTCACCTT
IGLC	F	CTCCAAACWGAGCAACAGCA
IGLC	R	CTTCACTGTCTTCKTCACGG
IGKC	F	TGTCGTGTGCTTGGTGAATG
IGKC	R	TTCTTGCTGTCCTGGTCTGT

ACTB, beta-actin

SDHA, succinate dehydrogenase complex flavoprotein subunit A

PPIA, peptidylprolyl isomerase A

Supplementary Table 2 – *IGHV* genomic features

IGHV gene	Fct	Octamer (promoter)	bp	ini.	L-PART1 (exon 1) (bp)	intron (bp)	splice sites	V-EXON (exon 2) (bp)	V-RS			Accession #	Start of V-EXON
									HEPTAMER	SPACER (bp)	NONAMER		
IGHV2-1	P[1]	-	-	-	-	-	-	282	CACAGTG	23	ACACCACCA	NC_030828	68,993,831
IGHV2-2	P[2]	GTGCACAT	107	ATG	46	78	TC/GG	305	CACAGTG	23	CACTAGCCA	NC_030828	68,976,671
IGHV1-3	F	ATGCAAAT	107	ATG	46	82	GT/AG	304	CACAGTG	23	ACAAAAACC	NC_030828	68,974,829
IGHV2-4	P[2]	GTGCACAT	107	ATG	46	86	GG/GG	305	CACAGTG	24	CACTAGCCA	NC_030828	68,963,254
IGHV1-5	F	ATGCAAAT	107	ATG	46	82	GT/AG	304	CACAGTG	23	ACAAAAACC	NC_030828	68,961,453
IGHV3-6	P[1]	-	-	-	-	-	-/GG	305	CACAGTG	23	CACTAGCCA	NC_030828	68,956,171
IGHV2-7	P[1]	-	-	-	-	-	-/AG	303	CACAGTG	23	CCAGAAACC	NC_030828	68,949,593
IGHV1-8	F	GTGCAAAT	107	ATG	46	82	GT/AG	304	CACAGTG	25	ACAAAAACC	NC_030828	68,946,570
IGHV1S9	P[3]	ATGCAAAT	107	ATG	46	71	GG/AG	304	CACAGTG	24	ACGAAAACC	LWLT01000331	3,435
IGHV1S10	P[4]	ATGCAAAT	105	ATG	46	71	TG/AG	295	CACAGTG	22	ACGAAAACC	LWLT01000331	31,721
IGHV1S11	P[5]	ATGCAAAT	107	ATG	46	71	TG/AG	304	CACAGTG	23	ACAAAAACT	LWLT01000361	29,484
IGHV3S12	P[1]	-	-	-	-	-	-/AG	303	CACAGTG	23	CCAGAAACC	LWLT01000361	24,203
IGHV1S13	P[4]	ATGCAAAT	103	ATG	46	71	TG/AG	304	CACAGTG	23	ACGAAAACC	LWLT01000364	6,201
IGHV3S14	P[1]	-	-	-	-	-	-/GG	305	CACAGTG	23	CACAAGTCA	LWLT01000364	22,043
IGHV1S15	F	ATGCAAAT	107	ATG	52	76	GT/AG	304	CACAGTG	23	ACAAAAACC	LWLT01000364	23,792
IGHV2S16	P[1]	-	-	-	-	-	-/AG	303	CACAGTG	23	CCAGAAACC	LWLT01000364	29,080
IGHV2S17	P[1]	-	-	-	-	-	-/AG	297	CACAGTG	23	ACACCAGCC	LWLT01000390	7,585
IGHV2S18	P[6]	GTGCACAT	107	GTG	46	86	GG/GG	303	GACAGTG	23	CACTAGCCA	LWLT01000390	20,777
IGHV1S19	P[5]	ATGCAAAT	107	ATG	46	71	TG/AG	304	CACAGTG	23	ACAAAAACT	LWLT01000390	22,571
IGHV3S20	P[1]	-	-	-	-	-	-/AG	303	CACAGTG	23	CCAGAAACC	LWLT01000390	27,850
IGHV2S21	P[6]	GTGCACAT	107	GTG	46	86	GG/GG	299	CACAGTG	23	CACTAGCCA	LWLT01000862	28,010
IGHV1S22	P[3]	ATGCAAAT	107	ATG	46	71	TG/AG	303	CACAGTG	19	CCAAAAACC	LWLT01000862	26,223
IGHV3S23	P[1]	-	-	-	-	-	-/AG	303	CACAGTG	23	CCACAAACC	LWLT01000862	20,940
IGHV1S24	P[3]	ATGCAAAT	107	ATG	46	71	TG/AG	303	CACAGTG	23	ACGAAAACC	LWLT01000862	12,431
IGHV3S25	P[1]	-	-	-	-	-	-/AG	304	CACAGTG	23	CCAGAAACC	LWLT01001482	19,957
IGHV2S26	P[3]	ATGGACAT	105	ATG	44	82	GG/AG	297	CACAGTG	23	ACACCAGCC	LWLT01001482	13,657
IGHV2S27	P[2]	GTGCACAT	106	ATG	46	87	GG/GG	307	CACAGTG	23	CACTAACCA	LWLT01001482	685
IGHV2S28	P[2]	GTGCACAT	107	ATG	45	86	GG/GG	291	CACAGTG	23	CACTAGCCA	LWLT01008200	1,691
IGHV1S29	P[1]	-	-	-	-	-	-/AG	300	CACAGTG	23	ACAAAAACT	LWLT01008200	3,471
IGHV3S30	P[1]	-	-	-	-	-	-/AG	301	CACAGTG	23	CCAGAAACC	LWLT01008200	8,740
IGHV2S31	P[1]	-	-	-	-	-	-/AG	297	CACAGTG	23	ACACCAGCC	LWLT01008318	11,961
IGHV2S32	P[1]	-	-	-	-	-	-/GG	302	CACAGTG	23	CACTAACCA	LWLT01010025	10,965
IGHV1S33	P[3]	ATGCAAAT	107	ATG	46	71	TG/AG	305	CACAGTG	19	CCAAAAACC	LWLT01010025	9,190
IGHV3S34	P[1]	-	-	-	-	-	-/AG	303	CACAGTG	23	CCAGAAACC	LWLT01010025	3,904

Fct functionality, *F* functional, *P* pseudogene

1; exon 1 is missing, frameshifts and stop codons in exon 2

2; multiple frameshifts and stop codons in exon 2

3; stop codon in exon 1, frameshifts and stop codons in exon 2

4; stop codon in exon 1

5; stop codon in exon 1, mutated first conserved cysteine (C23>F)

6; mutated start codon (M>V), frameshifts and stop codons in exon 2

	FR1-IMGT (1-26)		CDR1-IMGT (27-38)	FR2-IMGT (39-55)		CDR2-IMGT (56-65)	FR3-IMGT (66-104)				CDR3-IMGT (105-)
	A (1-15)	B (16-26)	BC	C (39-46)	C′ (47-55)	C′C″	C″ (66-74)	D (75-84)	E (85-96)	F (97-104)	FG
	1 15	16 26	27 38	39 46	47 55	56 65	66 74	75 84	85 96	97 104	105
	|.............|	|.........|	|..........|	|......|	|.......|	|........|	|.......|	|........|	|..........|	|......|	|.
IGHV1-3	QVQLQESGP.SLVKP	SQTLSLTCTVS	GFSL....TSYG	VGWVRQAP	GKALEWLGG	ISSG...GST	GYNPALK.S	RLSITRDTSK	SQVSLSLSSVTT	EDTAVYYC	AR
IGHV1-5	QVQLQESGP.SLVKP	SQTLSLTCTVS	GVSL....TSYA	VGWVRQAP	GKALEWLGI	IYSG...GST	YYNSALK.S	RLSITRDTSK	SQVSLSLSSVTT	EDTAVYYC	AR
IGHV1-8	QVQLQESGP.SLVKP	SQTLSLTCTVS	GFSL....TSYA	VGWVRQAP	GKALEWVGG	ISSG...GST	GYNPALK.S	RLSITRDTSK	SQVSLSLSSVTT	EDMALYYC	AR
IGHV1S10 P	QVQLQESGP.SLVKP	SQTLSLPCTDS	GFSL....TSCG	AGWVRQAP	GKRRSGLGY	HI......CG	TQASTLK.S	RLSITRDTSK	SQVSLSRSSVTT	EDTALYYC	AR
IGHV1S11 P	QVQLQESGP.SLVKP	SQTLSLTFTDS	GFSL....TSYG	AGWVRQAP	GKAPEWVGG	IDYD...GDT	SYNPALK.S	RLSITRDTSK	SQVSLSRSSVTT	EDTAVYYC	AR
IGHV1S13 P	QVQLQESGP.SLVKP	SQTLSLTCTDS	GFSL....TSYG	AGWVRQAP	GKAPEWVGG	IDYD...GDT	SYNPALK.S	RLSITRDTSK	SQVSLSRSSVTT	EDTALYYC	AR
IGHV1S15	QVQLQESGP.SLVKP	SQTLSLTCTDS	GVSL....TSYG	AGWVRQAP	GKALHWVGG	VDYD...GDT	SYNPALK.S	RLSITRDTSK	SQVSLSLSSVTT	EDTAVYYC	AR
IGHV1S19 P	QVQLQESGP.SLVKP	SQTLSLTFTDS	GFSL....TSYG	AGWVRQAP	GKAPEWVGG	IDYD...GDT	SYNPALK.S	RLSITRDTSK	SQVSLSRSSVTT	EDTAVYYC	AR

Supplementary Figure 1 Protein display of in-frame *IGHV* genes. Amino acid numbering is based on the IMGT system as discussed in the methods section. Where a specific residue is absent from the protein, the symbol (.) is used as a spacer in order to maintain correct numbering. The location of the CDR loops (BC, C’C”, and FG) and the framework beta-strands (A, B, C, C’, etc.) are indicated at top. Putative pseudogenes which have an in-frame variable region are indicated with a (*P*) beside the gene name

gene	5′D-RS			D-REGION	3′D-RS		
	NONAMER	SPACER	HEPTAMER		HEPTAMER	SPACER	NONAMER
IGHD1	GGTCCCAGC	AGGTGTGCATGT	CGCCGTG	AGAATACCATGATGATAGCTACTGCTATAGC	CACAGTG	GCTCAGGCCCTG	ACATAAAGC
IGHD2	GGTTTCTGA	TGCCGGCTGCAT	CATGGTG	GTAGTTGTTATAGTGGTTATGGTTATGGTTATGGTTATGGTTATGC	CACAGTG	ACACTCTCTGGG	ACAAAAACC
IGHD3	GGTTTGTGA	TGCCGGCTGCGT	CATGGTG	GTGATATGATAGGTATGGTTGTAGTTACTGTAGTATCGCTAC	CACAGTG	ACACTCTCAGTA	TTAGAAACC
IGHD4	GGTTTCTGA	TGCTGGCTGTGT	TATGGTG	GTAGTTATTATAGCGATTATGGTTATGC	CACAGTG	ACACTCTCTGGA	ACAAAAACC

Supplementary Figure 2 *IGHD* genomic features. Nucleotide sequence of the four goat *IGHD* genes, including features of both 5’ and 3’ recombination signals

gene	J-RS			J-REGION nt and AA sequences	splice site
	NONAMER	SPACER	HEPTAMER		
IGHJ1	GGGTTTCCA	TGCCCCCCAGCACAGGGCCAGCC	CACTGTG	ACTATGCTGACTTCCATCTCTGGGACCAGGGTGCCCTGGTCACCGTCTCCTCA<br>Y A D F H L W D Q G A L V T V S S	GG
IGHJ2	TGTTTTTGT	AGGAAAGAAGCAGCAGAAGAGA	AGCTGTG	CTGCTGGGACTTGGATCTCTGGGGCCAGCGCACCCCAGTCACCGCGTCCTTGAGCA<br>C W D L D L W G Q R T P V T A S L S	GT
IGHJ3	TGCTTTTGA	CTCCTGGGGCCAGCACGCCCCGGT	CACAGTT	TCCTCAGGTGAGACGGCTCTCTGCCCGCTCTGTCCTGGGCTGGGAAAGAAGTCTCCAGA<br>S S G E T A L C P L C P G L G K K S P	GG
IGHJ4	GGTTTTCGC	ACAGCCCCTAACGGGGCCCATGG	CACTGTG	ACTATATTGACTACTGGGGCCCAGGACTCCTGGTCACCGTCTCCTCAG<br>Y I D Y <u>W G P G</u> L L V T V S S	GT
IGHJ5	CGAGCTCGT	GCTTGGGGTCCCAGCATCACTGT	CACAGTG	TAACGACTGGCTCAAGCACTGGGGCCAGGGACCCTGGACACTGTCTGCTCA<br>+ R L A Q A L G P G T L D T V C S	GC
IGHJ6	GGTTTTTGT	TGGGCGAGGCTGGAGATAATCAC	CACTGTG	ATTACTACGGTGTAGATGTCTGGGGCCGAGGACTCCTGGTCACCGTCTCCTCAG<br>Y Y G V D V <u>W G R G</u> L L V T V S S	GT

Supplementary Figure 3 *IGHJ* genomic features. Nucleotide sequence and putative amino acid sequence of the six goat *IGHJ* genes, including features of the recombination signal and splice site. The canonical framework motif W/F-G-X-G is underlined where intact. The presence of a premature stop codon in *IGHJ5* is indicated with the symbol (+)

```
                      A                B           BC             C      CD     D         DE            E      EF   F             FG             G
                    (1-15)          (16-26)        (27-38)     (39-45)       (77-84)                 (85-96)     (97-104)     (105-117)      (118-128)
                ---------------->  ----------->                 ------->      -------->             ------------> --------->                  ----------->
                1           15   16       26   27        38   39   45       77    84              85        96  97   104   105       117   118     128
        87654321|.............|123|.........|   |..........|   |.....|1234567|......|12345677654321|..........|12|......|   |...........|   |.........|
IGKC        .SDAQPSVFLFKPSDEQLR...TGTVSVVCLVN   DFYP....KDIS   VKWKVDGVTQNS..NFQNSFTDQDSKK.....STYSLSSTLTLSSSEY..QSHNAYAC   EVSHKS..LTTAL   VKSFNKNEC
IGLC1       QPKSAPSVTLFPPSTEELN...ANKATVVCLIS   DFYP....GSVT   VA+KADGSTITR..NVETTQASKLSN......SKYAASSYLTLTGSEW..KSKSSYSC   EVTHE....GSTV   TKTVKPSECP
IGLC2       QPKSAPSVTLFPPSTEELN...ANKATVVCLIS   DFYP....GSVT   VVWKADGSTINQ..NVKTTQASKQSN......SKYAASSYLTLTGSEW..KSKSSYSC   EVTHE....GSTV   KKTVKPSECS
```

Supplementary Figure 4 Protein display of *IGKC* and *IGLC* genes. Numbering and features are as described in the methods section and Supplementary Figure 1. A premature stop codon (i.e. position 41 in *IGLC1*) is indicated with the symbol (+)

gene	J-RS			J-REGION nt and AA sequences	splice site
	NONAMER	SPACER	HEPTAMER		
IGLJ1	GGTTTTGGT	CCGAGGCTCAGT	CACTGTG	CTTCGTCTTAGGTGGCGGGACCCAGCTCACCGTCCTAG<br>F V L G G G T Q L T V L	GT
IGLJ2	GGTTTTTGT	GCCAGCCTGTGT	CACTGTG	TGGTGTTTTCGGCAGCGGGACCAGGCTGACCGTGCTGG<br>G V <u>F G S G</u> T R L T V L	GT

Supplementary Figure 5 *IGLJ* genomic features. Nucleotide sequence and putative amino acid sequence of both goat *IGLJ* genes, including features of the recombination signal and splice site. The canonical framework motif W/F-G-X-G is underlined where intact

Supplementary Table 3 – *IGLV* genomic features

IGLV gene	Fct	Octamer (promoter)	bp	ini.	L-PART1 (exon 1) (bp)	intron (bp)	splice sites	V-EXON (exon 2) (bp)	V-RS			Start of V-EXON (Accession: NC_030824)
									HEPTAMER	SPACER (bp)	NONAMER	
IGLV3-1	P[1]	ATTGTCAT	83	ATG	46	368	GT/AG	293	CACAGTG	23	CCCAAACCC	212,969
IGLV3-2	F	ATTTGCAT	108	ATG	46	144	GT/AG	299	CACAGTG	23	ACACAAACC	217,287
IGLV3-3	F	ATTTGCAT	107	ATG	46	159	GT/AG	302	CACAGTG	23	ACAGAAACC	222,045
IGLV3-4	F	ATTTGCAT	84	ATG	46	145	GT/AG	299	CACAGTG	23	ACACAAACC	230,423
IGLV(V)-5	P[2]	-	-	-	-	-	-/AG	328	CACAGGG	23	GCATACCCC	234,277
IGLV3-6	P[3]	ATTTACAT	107	ATG	46	145	GT/AG	212	-	-	-	237,612
IGLV3-7	F	ATTTGCAT	84	ATG	46	154	GT/AG	299	CACAGTG	23	ACACAAACC	243,387
IGLV3-8	F	ATTTGCAT	84	ATG	46	154	GT/AG	299	CACAGTG	23	ACACAAACC	248,206
IGLV3-9	P[1]	ATTTGCAT	107	ATG	46	144	GT/AG	298	CACAGTG	23	ACACAAACC	255,357
IGLV2-10	F	ATTTGCAT	97	ATG	46	115	GT/AG	308	CACAGTG	23	ACCAAAACC	263,989
IGLV2-11	F	ATTTGCAT	97	ATG	46	116	GT/AG	308	CACAATG	23	ACCAAAACC	287,348
IGLV2-12	P[4]	ATTTGCAT	97	GTG	46	114	GT/AG	308	CACAGTG	23	ACCAAAACC	302,229
IGLV2-13	P[4]	ATTTGCAT	97	GTG	46	114	GT/AG	308	CACAGTG	23	ACCAAAACC	309,819
IGLV2-14	P[5]	ATTTGCAT	97	ATG	46	113	GT/AG	308	CACAGTG	23	GCCAAACCC	313,327
*IGLV-15**	P[6]	ATTTGTGT	95	ATG	46	-	GT/-	-	-	-	-	317,960
IGLV1-16	P[7]	CTTTGAAT	104	ATG	45	109	GT/AG	300	CCCACTG	23	ACAAAGACC	411,979
IGLV1-17	F	ATTTGCAT	104	ATG	46	108	GT/AG	310	CACAGTG	23	ACAAAAACC	417,143
IGLV1-18	F	ATTTGCAT	107	ATG	46	109	GT/AG	301	CACAGTG	23	ACAAAAACC	434,558
IGLV5-19	P[1]	ATTTGTAC	88	ATG	46	1031	GT/AG	327	CACGGTG	23	ATCAAAACA	438,626
IGLV1-20	F	ATTTGCAT	107	ATG	46	108	GT/AG	307	CACGGTG	23	GCAAAAGCC	450,996
IGLV8-21	P[1]	ATTTGCAT	102	ATG	46	97	GT/AG	304	CGCAGTG	23	ACCAAAACC	454,265
IGLV5-22	P[8]	ATTTGCAT	87	GTG	46	130	GT/AG	307	CAGATGA	12	ACAAAAACC	459,885
IGLV1-23	P[1]	ATTTGCAT	107	ATG	46	110	GT/AG	307	CACGGAG	23	ACAAAAACC	475,878
IGLV8-24	P[1]	ATTTGCAT	110	ATG	37	97	GA/AG	310	CGCAGTG	23	ACTAAAACC	483,773
IGLV5-25	P[9]	-	-	-	-	-	-/AG	304	CACAGCG	23	ACGAGAGCC	489,083
IGLV1-26	F	ATTTGCAT	107	ATG	46	107	GT/AG	307	CACAGTG	23	ACAAAAACC	489,925
IGLV1-27	P[10]	CTTTGCGA	101	ATC	46	124	CA/AG	301	CACACTG	22	ACAAAGACC	493,241
IGLV8-28	P[11]	ATTTGCAT	101	GCG	46	97	GT/AG	298	CGCAGTG	23	GCCCAAACC	495,363
IGLV8-29	P[1]	GTTTGCAT	81	ATG	46	98	GT/AG	309	CCCAGTG	23	AACAAAACC	503,044
IGLV1-30	F	TTTTGCAT	108	ATG	46	107	GT/AG	310	CACAGTG	23	ACAAAAACC	509,454
IGLV(V)-31	P[12]	CATTGTAT	79	TCG	49	110	GT/AG	324	CACAGTG	21	AGAAACCCC	513,632
IGLV8-32	P[1]	ATTTGCAT	100	ATG	46	97	GT/AG	312	CACAGTG	23	ACTAAAACC	522,876
IGLV1-33	F	ATTTGCAT	104	ATG	46	109	GT/AG	310	CACAGTG	23	ACAAAACCT	556,145
IGLV1-34	P[13]	-	-	ATG	46	110	GT/AG	303	CACACTG	22	ACAAAGACC	552,517
IGLV5-35	P[9]	-	-	-	-	-	-	274	CAACAGG	23	ACGGGAAAC	551,888
IGLV1-36	F	ATTTGCAT	107	ATG	46	109	GT/AG	307	CACAGTG	23	ACGAAAACC	549,592
IGLV5-37	P[2]	-	-	-	-	-	-/AG	327	CATGGTG	23	ACAGAAACC	545,604
IGLV1-38	P[1]	ATTTGCAT	107	ATG	46	108	GT/AG	305	CACAGTG	23	GCAAAAGCC	540,991
IGLV8-39	P[1]	GATTGCAT	102	ATG	46	97	GT/AG	304	CGCAGTG	23	ACCAAACCC	537,744
IGLV5-40	P[2]	-	-	-	-	-	-	319	CAGCCTG	23	CGTCAAATC	533,180
IGLV1-41	F	ATTTGCAT	107	ATG	46	109	GT/AG	310	CACAGTG	23	ACAAAAACC	532,312
IGLV1-42	P[1]	CTTTGAAT	104	ATG	46	109	GT/AG	302	CCCAGTG	23	ACAAAGACC	528,740
IGLV1-43	P[1]	unknown	>78	ATG	47	109	GT/AG	309	CACAGTG	23	ACAAAAACC	523,595
IGLV(V)-44	P[12]	CATTGTAT	79	TCG	49	110	GT/AG	310	CACAGTG	19	ACAGAAACC	562,851
IGLV8-45	F	ATTTGCAT	102	ATG	46	99	GT/AG	310	CACAGTG	23	ACTAAAACC	572,182
IGLV1-46	F	ATTTGCAT	107	ATG	46	107	GT/AG	310	CACAGTG	23	ACAAAAACG	578,333
IGLV1-47	P[7]	ATTTGCAT	105	ATG	45	109	GT/AG	303	CACACTG	22	ACAAAGACC	582,982
IGLV5-48	F	ATTTGCAT	88	ATG	46	118	TA/AG	328	CACGGTG	23	ACAGAAACC	589,134
IGLV1-49	F	ATTTGCAT	104	ATG	46	107	GT/AG	307	CACAGTG	23	ACGAAAACC	593,240
IGLV1-50	F	ATTTGCAT	98	ATG	46	109	GT/AG	310	CACAGAG	23	ACGAAAGCC	600,330
IGLV5-51	P[1]	ATTTGCAT	89	GTG	45	118	GT/AG	330	CCCGGTG	21	ACACAAAGA	604,402
IGLV1-52	F	ATTTGCAT	107	ATG	46	109	GT/AG	310	CACAGTG	22	ACAAAAACC	607,665
IGLV1-53	F	ATTTGCAT	104	ATG	46	109	GT/AG	310	CACAGTG	23	ACAAAAACC	616,478
IGLV5-54	P[2]	-	-	-	-	-	-/AG	314	CACTGGT	-	-	618,853
IGLV(V)-55	P[1]	CTTTGAAT	78	ATG	49	110	GT/AG	309	CACAGTG	21	AGAAACCCC	621,317
IGLV8-56	P[1]	ATTTGCAT	102	ATG	46	99	GT/AG	309	CACAGTG	23	ACTAAAACC	630,796
IGLV8-57	P[9]	-	-	-	-	-	-	228	-	-	-	635,129
IGLV1-58	F	ATTTGCAT	108	ATG	46	109	GT/AG	310	CACAGTG	23	ACAAAACCT	637,563
IGLV1-59	P[1]	GTTTGAAT	105	ATG	46	110	GT/AG	303	CCCAGTG	19	ACAAAGACC	640,871
IGLV5-60	F	ATTTGCAT	89	ATG	46	118	GT/AG	328	CACGGTG	23	ACAGAACAA	645,084
IGLV1-61	F	ATTTGCAT	107	ATG	46	109	GT/AG	304	CACGGTG	23	ACAAAAACC	647,866
IGLV5-62	P[14]	ATTTGCAT	88	ATG	45	118	GT/AG	325	CCAGGTG	21	ACAGGCACT	652,760
IGLV(III)-63	P[9]	-	-	-	-	-	-	204	-	-	-	657,169

Fct functionality, *F* functional, *P* pseudogene; *ini* initiation codon
*; accession coordinate is for translation initiation site in exon 1
1; frameshifts and stop codons in V-EXON
2; exon 1 is missing, multiple frameshifts and stop codons in V-EXON
3; truncated V-EXON with frameshifts and stop codons
4; mutated start codon (M>V)
5; stop codon at position 1 of V-EXON
6; V-EXON is missing
7; stop codon in exon 1, frameshifts and stop codons in V-EXON
8; mutated start codon (M>V), frameshifts and stop codons in V-EXON
9; exon 1 is missing, truncated V-EXON with frameshifts and stop codons
10; mutated start codon (M>I), multiple frameshifts and stop codons in V-EXON
11; mutated start codon (M>A), frameshifts and stop codons in V-EXON
12; mutated start codon (M>S), frameshifts and stop codons in V-EXON
13; octamer missing, frameshifts and stop codons in V-EXON
14; frameshift in exon 1

```
                        FR1-IMGT                    CDR1-IMGT        FR2-IMGT           CDR2-IMGT                       FR3-IMGT                           CDR3-IMGT
                         (1-26)                      (27-38)          (39-55)            (56-65)                        (66-104)                           (105-117)
                     A                B                BC           C          C′            C′C″         C″          D               E              F           FG
                   (1-15)          (16-26)                       (39-46)   (47-55)                     (66-74)     (75-84)         (85-96)        (97-104)
              -------------->  ----------->                 ------->  --------->             --------->  ---------->  ------------->  ------->
              1            15  16        26  27         38  39    46  47      55  56      65  66     74  75      84  85          96  97   104  105   113
              |.............|  |.........|  |..........|  |......|  |.......|  |.........|  |.......|  |........|  |..........|  |......|  |.......|
IGLV1-17      QAVLTQPSS.VSKSL  GQSVSITCSGS  SSNVG...YGNY  VSWYQQVP  GSAPKLLIY  GA.......T  SRASGVP.A  RFSGSR..SG  NTATLSISSLQA  EDEADYYC  ASYDSSSYN
IGLV1-18      QAVLTQPSS.VSGSP  GQRVSITCSGS  NIG......SSG  VGWYQQLP  GSGLKTVIY  YN.......S  NRPSGVP.D  RFSGSK..SG  NTATLTISSLQA  EDEADYYC  GSYDSSSYN
IGLV1-20      QAVLTQPSS.VSRSL  GQRVTITCSGS  SSNT....GGNF  VGWCQQLP  GMAPKTLIY  GD.......S  NRPSGVP.G  WFSGSK..SG  NSASMTIASLQA  KEEAGYYC  LSWDDSLNC
IGLV1-26      QAVLTQPPS.VSGSP  GQSVSITCTGS  SSNI....GRYG  VAWYQQLP  GSAPKLLIY  CT.......T  SGALGSS.D  RFSCSM..SG  NTATLTISSLQA  EDEADYYC  ATYDSSSYN
IGLV1-30      QAVLTQPSS.VSRSL  GQRVSITCSGS  SSNVG...YGNY  VGWYQQVP  GSGLRTLIY  GA.......T  SRASGVP.D  RFSSSK..SG  NTATLTISSLQA  EDEADYFC  SSYDSSGYN
IGLV1-33      QAVLTQPSS.VSRSP  GQRVFITCSGS  SSNVG...TGNY  VGWYQQVP  GSAPKLLIY  GA.......T  SRASGVP.D  RFSGSR..SG  NTATLTISSLQA  EDEADYYC  SSYQSGYSY
IGLV1-36      QAVLTQPPS.VSGSP  GQRVSITCSGS  SSNI....GSSG  VAWFQQLP  GSGLRIVIY  YS.......S  NRPSGVP.D  RFSGSR..SG  NTATLTISSLQA  EDEADYYC  ASYQSGYSY
IGLV1-41      QAVLTQPPS.VSGSP  GQRVSITCSGS  SSNIG...GGNY  VSWYQQLP  GSAPKLLIY  CT.......S  SRASGVP.D  RFSGSR..SG  NTATLTISSLQA  EDEADYYC  ATYESSSYN
IGLV1-46      QAVLTQPPS.VSGSP  GQRVSITCTGS  SSNIG...GGNY  VGWYQQLP  GSGLRTIIY  ST.......S  SRPSGVP.D  RFSGSR..SG  NTATLTISSLQA  EDEADYYC  ATYESSSYN
IGLV1-49      QAVLTQPSS.VSGSL  GQRVSITCSGS  SSNI....GRGY  GSWYQQVP  GSAPKLLIY  GA.......T  SRASGVP.D  RFSGSR..SG  NTATLTISSLQA  EDEADYYC  AAYDSSSSN
IGLV1-50      QAVLTQPSS.VSRSL  GQSVSITCSGS  SSNIG...GGYY  VGWYQQIP  GSAPRTLIY  QN.......S  KRPTGVP.N  RFSGSK..SG  STATLTISSLQA  EDEADYYC  SAYDSSISA
IGLV1-52      QAVLTQPSS.VSRSP  GQRVFITCSGS  SSNVG...TGNY  VGWYQQVP  GSAPKLLIY  GA.......T  SRASGVP.D  RFSGSR..SG  NTATLTISSLQA  EDEADYYC  SSYQSGYSY
IGLV1-53      QAVLTQPSS.VSKSL  GQSVSITCSGS  SSNVG...YGNY  VGWYQQVP  GSAPKLLIY  GA.......T  SRASGVP.A  RFSGSR..SG  NTATLSISSLQA  EDEADYYC  ASYDSSGYN
IGLV1-58      QAVLTQPPS.VSASL  GQRVSIFCNGS  SSNIG...GGNY  VGWYQLIP  GSGLRTIIY  GT.......T  GRPSGVL.D  RFSGSR..SG  NTATLTITSLQA  EDEVDYYC  ATYDSSSYN
IGLV1-61      HAVLTQPPS.VSGSL  GQSVTISCSGS  SSNI....GILG  VSWYQQLQ  GSAPKTLIY  GS.......N  KRPSGVP.D  RFSGTK..SG  NTGTLTITSLQA  EDEADYYC  ASADLILNS
IGLV2-10      QSGLTQPAS.VSGNP  GQTVTISCTGT  SSDIG...SYNG  VGWYQQLP  GSAPKTLIY  NL.......N  KRPSGIP.A  RFSGSK..SG  NTATLTISGLQA  EDEADYYC  SSYKSGGTV
IGLV2-11      QSALTQPAS.VSGNP  GQTVTISCTGT  SSDIG...GYNY  IGWYQQLP  GSAPKTLIY  NL.......N  KRPSGIP.A  RFSGSK..SG  NTATLTISGLQA  EDEADYYC  SSYKSGGTV
IGLV2-12 P    QSALTQPAS.VSGNP  GQTVTISCTGT  SSDIG...GYNY  VGWYQQLP  GSAPKTLIY  EV.......S  KRPSGIP.A  RFSGSK..SG  NTATLTISGLQA  EDEADYYC  SSPKSGYTV
IGLV2-13 P    QSALTQPAS.VSGNP  GQTVTISCTGT  SSDIG...GYNY  VGWYQQLP  GSAPKTLIY  NV.......N  NRPSGIP.A  RFSGSK..SG  NTATLTISGLQA  EDEADYYC  SSPRSGYTV
IGLV2-14 P    +SGLTQPAS.VSGNP  GQTVTISCTGT  SSDIG...GYNY  VGWYQQLP  GSAPKTLIY  NV.......N  NRPSGIP.A  RFSGSK..SG  NTATLTISELQA  EDEADYYC  SSARSGHTV
IGLV3-2       SSQLTQPPA.VSVSL  GQTASITCQGD  DLA......FLS  ANWYQLKP  GQAPVTVIY  GG.......S  DRASGIP.D  RFSGSK..SD  TTATLTIRGAQA  EDEADYYC  QSGGIDVDA
IGLV3-3       SYELTQPTS.VSVVL  GQTAKVTCSGD  LLD......EQY  TQWHQQKP  GQAPELVIY  ED.......S  KRRSGIP.D  RFSGSS..SS  KTAILTISGVRA  EDEADYYC  VSWDSGNYNI
IGLV3-4       SYELTQPTS.VSVAL  GQTAKITCQGD  LLD......EKY  TAWYQQKP  GQAPVKVIC  KD.......S  ERPSGIL.D  RFSGSS..SG  KTATLTISGART  EDEADYYC  LSADSSNNP
IGLV3-7       SYELTQPTS.VSVAL  GQTAKVTCSGD  LLD......ENF  AHWYQQKP  GQAPVLVIY  LN.......S  ERASGIP.D  RFSGSS..SG  STATLTISGVQA  EDEADYFC  QSYDSSNNP
IGLV3-8       SYELTQPTS.VSVAL  GQTAKVTCQGD  NLG......SSY  VQWHQQKP  GQAPVTVIY  QD.......S  KRPSGIP.D  RFSGSN..SG  NTATLTISGART  EDEADYYC  QSADSSNNP
IGLV5-48      QPVLTQPAS.LSASP  VTSARLSCTLS  SGYNV...GDFS  LSWFQQKP  GSPPWYVVR  VKSD...SDK  NQSSGVP.S  NFFGSKDASA  NAGLLLISGLQP  EDEADYYC  AVWHGDTNA
IGLV5-60      QPLLTQPAS.LSASP  GASARLTCTLS  SGYNV...GNYS  IYWYQQKA  GSPPRYLLR  FKSD...SDK  HQGSGVP.N  RFSGSKDAST  NAGLLLISEVQP  EDEADYYC  AVWHGDTNA
IGLV5-62 P    QPVLTQPAS.LSASP  GASARLSCTLS  SGYSA...GSYQ  MSCFQQKP  GGPPQYLLM  FKSH...SDK  PQGSRVP.S  HFSGSKAASA  NTELLLISGLQT  PDEADCNC  YCHQNTGI
IGLV8-45      AQTVIQEPA.LSVSP  GGTVTLACALS  SGSVT...TYNE  PSWYQQTP  GQVPRNVIY  NT.......N  TRASGVP.D  RFSASI..SG  NKATLTITGAQP  EDEADYHC  LLYQGSGSYS
```

Supplementary Figure 6 Protein display of in-frame *IGLV* genes. Numbering and features are as described in the methods section and Supplementary Figure 1. A premature stop codon (i.e. position 1 of *IGLV2-14*) is indicated with the symbol (+)

Supplementary Table 4 – *IGKV* genomic features

IGKV gene	Fct	Octamer (promoter)	bp	ini.	L-PART1 (exon 1) (bp)	intron (bp)	splice sites	V-EXON (exon 2) (bp)	V-RS HEPTAMER	V-RS SPACER (bp)	V-RS NONAMER	Start of V-EXON (Accession: NC_030818)
IGKV1-1	F	ATTTGCAT	91	ATG	49	125	GA/AG	302	GTGTCAC	12	AAACCACCC	46,904,452
IGKV1-2	F	ATTTGCAT	95	ATG	56	206	GT/AG	293	CACTGTT	12	ATAAACAGT	46,895,815
IGKV1-3	F	ATTTGCAT	93	ATG	52	123	GT/AG	298	CACAGTG	12	ACATAAGCC	46,880,251
IGKV(II)-4	P[1]	-	-	-	-	-	-	267	TACTGCT	8	GCAAAAACT	46,871,611
IGKV1-5	P[2]	ATTTGCAG	93	ATG	49	114	GT/AG	289	CCCTCTG	12	ACAAAATCC	46,864,699
IGKV1-6	P[2]	ATTTGCAT	93	ATG	52	527	TG/AG	295	TACAGTG	12	ACATAAACC	46,854,461
IGKV2-7	F	ATTTGCAT	100	ATG	49	345	GT/AG	313	CACAGTG	12	ACACAAACC	46,850,568
IGKV2-8	F	ATTTGCAT	100	ATG	49	390	GT/AG	313	CACAGTG	12	ACAGAAACC	46,841,338
IGKV2-9	F	ATTTGCAT	100	ATG	49	412	GT/AG	315	CACAGTG	12	ACACAAACC	46,835,433
IGKV2-10	F	ATTTGCAT	99	ATG	49	413	GT/AG	313	CACAGTG	12	ACACAAACC	46,809,111
IGKV2-11	F	ATTTGCAT	100	ATG	49	389	GT/AG	313	CACAGTG	12	ACATAAACC	46,797,570
IGKV1-12	P[3]	ATTTACAT	95	ATA	50	131	AG/AG	291	CACAGTA	12	ACATAAACC	46,790,983
IGKV2-13	P[2]	ATTTGCAC	100	ATG	49	387	CC/AG	312	CACAGTG	12	ATACAAATC	46,784,160
IGKV3-14	P[4]	-	-	-	-	-	-/AG	296	TCGAGTG	12	ACAAAAACC	46,776,180
IGKV1-15	P[5]	GATTACAT	83	ATG	49	132	GG/AG	292	CACAGTG	12	AAATGGCAA	46,771,640

Fct functionality, *F* functional, *P* pseudogene; *ini* initiation codon

1; exon 1 is missing, multiple frameshifts, stop codons in V-EXON which is also truncated

2; frameshifts, stop codons in V-EXON

3; mutated start codon (M>I), stop codon at position 103

4; exon 1 is missing, multiple frameshifts, stop codons in V-EXON

5; stop codon in L-PART1

	FR1-IMGT (1-26)		CDR1-IMGT (27-38)	FR2-IMGT (39-55)		CDR2-IMGT (56-65)	FR3-IMGT (66-104)				CDR3-IMGT (105-117)
	A (1-15)	B (16-26)	BC	C (39-46)	C′ (47-55)	C′C″	C″ (66-74)	D (75-84)	E (85-96)	F (97-104)	FG
	1 15	16 26	27 38	39 46	47 55	56 65	66 74	75 84	85 96	97 104	105 113
IGKV1-1	DIQVTQSPSSLSACL	GDRVSITCQAS	QNI......DTK	LAWYQQKP	RKAPKLLIY	AV.......S	RSPSWFP.S	QFSGSG..FG	IDFILTISSLKA	DDIATYYC	QQDHGLPPT
IGKV1-2	EAVLYQTPAYIAVPL	GESISITCRAN	QSI......SDY	LSWHKQKP	GQAPMILIY	DA.......D	NRRNGVP.E	RFTATQ..SG	TDFVFTISQIEA	DDAAMYYC	QQSYVFPP
IGKV1-3	DIQVTQSPSSLSASL	TERVSITCRTS	QSV......SNY	LNWYQQKP	GQAPKLLIY	YA.......T	RLHTDVP.S	RFSGSG..SG	TDYTLTISSLEA	DDAATYYC	LQGYSTP
IGKV1-12 P	DLQMIQSLSSLSASL	GDRVSITCQAS	QSV......RNN	LQWYQEKP	GKAPEFLFC	DT.......T	SVHTGVP.S	RFSGSG..SG	TDYAFTTSSLEA	DDFAAY+C	KQENSRP
IGKV1-15 P	DLQMTQSPSSPFASL	GDAITWQASQS	IS........KY	LAWYQQKP	GKAPKLLIF	RA.......S	RLHTGVP.S	WFSGSG..SG	TDYTLTISELEA	DDVATYYC	QQYNSIP
IGKV2-7	DVVLTQTPLSLSVIP	GETASISCKSS	QSLVHS.DGKTY	LNWIKHKP	GQSPEGLIY	QV.......S	NRYSGVS.D	RLTGSG..SG	TDFTFTISRVQA	EDAGVYYC	YQGTEAP
IGKV2-8	AMMQTQTLRSLSVIH	GEKASISCRAS	QSIQNR.YGYNF	LHWYVQKP	SQSPQLLIY	RA.......S	NWESGVP.D	RFTSSG..LG	ADTILIVSRVEA	EDARVYYC	QQSLQAP
IGKV2-9	DIVLTQTPLSLSVIP	GGTVSISCKSS	QSLKYS.DGKTY	LSWFQHKP	GQSPRRLIY	QV.......S	NRNTGVP.D	RFTGSG..AE	TDFTLPISSVQA	EDAGVY.C	SFQATYYDP
IGKV2-10	DVVLTQTPLSLSVIP	GGTASISCKSS	QSLKYS.DGNTY	LYWFQHKP	GQSPRRLIY	LV.......S	NRDTGVP.D	RFTGSG..AE	TDFTLTISSVQA	EDAGVYYC	FQAINYP
IGKV2-11	AIVLTQTPRSLSVIP	GETASISCRAS	QSVQNR.YGDNF	LHWYVQKP	SQSPQLLIY	AA.......S	NRASGVS.D	RFTGSG..SG	TDFTLKISRVEA	EDAGVYYC	QQSKETP

Supplementary Figure 7 Protein display of in-frame *IGKV* genes. Numbering and features are as described in the methods section and Supplementary Figure 1. A premature stop codon (i.e. position 103 of *IGKV1-12*) is indicated with the symbol (+)
